# Striatal dopamine transporter binding in adults with 22q11.2 copy number variants: a SPECT study

**DOI:** 10.1093/braincomms/fcag395

**Published:** 2026-10-19

**Authors:** Jeltje C C Spapens, Carmen F van Hooijdonk, Rik Schalbroeck, Amy L Sylvester, Erik Boot, Thérèse A M J van Amelsvoort, Claudia Vingerhoets, Jan Booij

**Affiliations:** Mental Health and Neuroscience Research Institute, Maastricht University, 6229 ER Maastricht, The Netherlands; Mental Health and Neuroscience Research Institute, Maastricht University, 6229 ER Maastricht, The Netherlands; Institute of Education and Child Studies, Leiden University, 2333 AK Leiden, The Netherlands; Department of Radiology and Nuclear Medicine, Amsterdam University Medical Centers, University of Amsterdam, 1105 AZ Amsterdam, The Netherlands; Mental Health and Neuroscience Research Institute, Maastricht University, 6229 ER Maastricht, The Netherlands; Mental Health and Neuroscience Research Institute, Maastricht University, 6229 ER Maastricht, The Netherlands; Advisium, ‘s Heeren Loo Zorggroep, 3818 LA, Amersfoort, The Netherlands; The Dalglish Family 22q Clinic, University Health Network, Toronto, Ontario M5G 2C4, Canada; Mental Health and Neuroscience Research Institute, Maastricht University, 6229 ER Maastricht, The Netherlands; Mental Health and Neuroscience Research Institute, Maastricht University, 6229 ER Maastricht, The Netherlands; Advisium, ‘s Heeren Loo Zorggroep, 3818 LA, Amersfoort, The Netherlands; Department of Radiology and Nuclear Medicine, Amsterdam University Medical Centers, University of Amsterdam, 1105 AZ Amsterdam, The Netherlands

**Keywords:** 22q11.2 deletion, 22q11.2 duplication, dopamine transporter, SPECT, Parkinson’s disease

## Abstract

Copy number variants at the 22q11.2 chromosomal locus, hemizygous 22q11.2 deletions or duplications, are associated with a broad spectrum of neuropsychiatric and other medical conditions. Individuals with a 22q11.2 deletion are at increased risk for developing early-onset Parkinson’s disease. The *catechol-O-methyltransferase* gene at 22q11.2 is involved in dopamine metabolism. While previous studies have examined several aspects of dopamine function in individuals with 22q11.2 copy number variants, striatal dopamine transporter availability, a regulator of synaptic dopamine, has not yet been systematically investigated. Therefore, dopamine transporter availability was quantified in nine adults with a 22q11.2 deletion, nine with a 22q11.2 duplication and eight controls using a brain-dedicated single-photon emission computed tomography (SPECT) system. Specific striatal binding ratios were calculated with the occipital lobe as the reference region. Permutation analysis of covariance controlling for age and sex showed significantly higher striatal dopamine transporter availability in adults with 22q11.2 deletions compared with those with duplications (*P* = 0.026, *η*^2^_p_ = 0.30). No significant differences were observed between adults with 22q11.2 copy number variants and controls. These findings suggest distinct nigrostriatal dopaminergic profiles associated with 22q11.2 copy number variant status, which may play a role in shaping frequently occurring neurological and psychiatric phenotypes in these individuals.

## Teaser

Spapens *et al*. report that adults with a 22q11.2 deletion show significantly higher striatal dopamine transporter availability than those with a 22q11.2 duplication, measured by single-photon emission computed tomography. These distinct nigrostriatal dopaminergic profiles may contribute to the neurological and psychiatric phenotypes associated with 22q11.2 copy number variants.

## Graphical Abstract

**Graphical Abstract:**
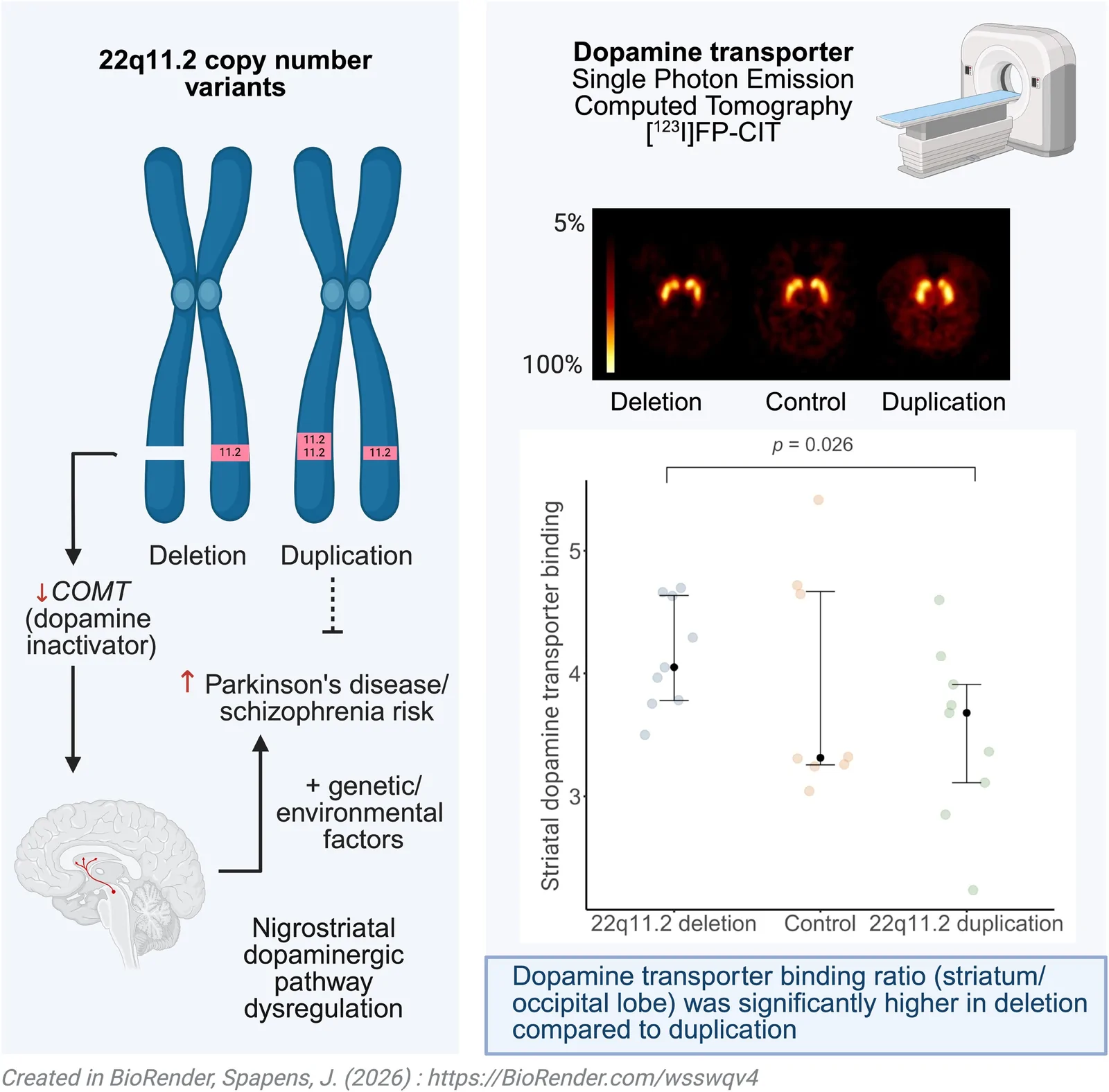

## Introduction

Copy number variants (CNVs) at chromosome 22q11.2 are among the most common pathogenic CNVs in humans, with 22q11.2 deletion affecting ∼1 in 2000 live births,^1-3^ and 22q11.2 duplication affecting ∼1 in 1600 live births.^1^ Both deletion and duplication are associated with a range of neuropsychiatric and medical conditions,^2^ where 22q11.2 deletion is generally associated with a relatively higher burden of medical comorbidities.^4^ CNVs at 22q11.2 have been associated with dopamine-related neuropsychiatric phenotypes, including attention-deficit/hyperactivity disorder (ADHD) during childhood and schizophrenia in (young) adulthood,^5,6^ and schizophrenia appears to be more strongly associated with deletions than duplications.^7,8^ Additionally, 22q11.2 deletion confers a markedly increased risk for Parkinson’s disease compared to both 22q11.2 duplication and the general population, especially at a young age of onset (Parkinson’s disease with an age of onset <50).^4^ A recent multicentre study reported 14% of the individuals with a 22q11.2 deletion over 50 years (*n* = 13/93) were affected by Parkinson’s disease and 23.7% had suspected Parkinson’s disease (*n* = 22/93), with a median age at motor onset of just 45 years.^9^ The elevated risk of Parkinson’s disease and parkinsonism is consistent with observations from earlier smaller cohorts.^10,11^ To the best of our knowledge, in 22q11.2 duplication, no cases of early-onset Parkinson’s disease have been reported.

In 22q11.2 deletion, hemizygous deletions of *catechol-O-methyltransferase* (*COMT*) may increase dopamine levels due to reduced metabolism.^12^ In a previous study in lymphoblasts, individuals with a 22q11.2 deletion showed roughly a 50% reduction in *COMT* mRNA, COMT protein, and enzyme activity as compared to healthy controls.^13^ Subsequently, elevated urinary and plasma dopamine levels were observed in young individuals with a 22q11.2 deletion (mean age 27.3 ± 7.0 years) compared to controls,^14^ but no significant difference in striatal D_2_ receptor binding.^15^ Moreover, reduced frontal dopamine D_2/3_ receptor binding has been reported in adults with a 22q11.2 deletion (mean age 38.1 ± 15.6 years), suggesting a compensatory mechanism for elevated dopamine levels in the prefrontal cortex.^16^ Conversely, reciprocal 22q11.2 duplication may exert opposing effects of gene dosage,^17^ as previously shown for opposing dopaminergic synthesis capacity^18^ and *COMT* mRNA levels compared to 22q11.2 deletion.^19^ Beyond *COMT*, several deleted genes in the 22q11.2 region are linked to oxidative stress and mitochondrial dysfunction,^20^ which are well-established features both in the 22q11.2 deletion models^21,22^ and idiopathic Parkinson’s disease patients.^23^ Together, chronically elevated dopamine and cellular stressors may possibly increase susceptibility to nigrostriatal degeneration in 22q11 deletion, subsequently contributing to the higher risk of Parkinson’s disease in adults.^10^

The findings of these previous studies highlight the importance of early detection and monitoring of parkinsonian symptoms in this population. Single-photon emission computed tomography (SPECT) imaging of the dopamine transporter (DAT), a key regulator of synaptic dopamine,^11,24^ may help distinguish Parkinson’s disease from non-degenerative parkinsonism in this population.^25^ However, striatal DAT availability has not yet been studied systematically in individuals with 22q11.2 CNVs. Given the elevated risk of (early-onset) Parkinson’s disease in 22q11.2 deletion, examining DAT availability may help to characterize dopaminergic function in this population. Including 22q11.2 duplications allows us to investigate whether dopaminergic alterations are specific to the deletion. In this study, we investigated striatal DAT availability in individuals with 22q11.2 CNVs, and healthy controls using dopamine transporter single-photon emission computed tomography (DaT-SPECT).

We hypothesized that individuals with 22q11.2 deletions would show increased striatal DAT availability compared with healthy controls, potentially reflecting reduced dopamine metabolism and previously reported hyperdopaminergic states, whereas individuals with 22q11.2 duplications would not exhibit the same pattern.

## Methods

The study protocol was approved by the medical-ethical review committee of Maastricht University (METC21-043/NL77267.068.21) and conducted in accordance with the Declaration of Helsinki. Data were collected between November 2021 and October 2025.

### Participants

The sample consisted of nine adults with a 22q11.2 deletion, nine adults with a 22q11.2 duplication, and eight healthy controls. Patients with 22q11.2 CNVs were recruited via Stichting Steun 22Q11, the Dutch Family Organization for 22q11.2 CNVs, the Dutch Adult 22q11 Expertise Center at Maastricht University Medical Centre (MUMC), and ‘s Heeren Loo Zorggroep (Amersfoort, The Netherlands). Healthy controls were recruited via advertisements on the internet. Participants were considered eligible for inclusion in the study if they were between 16 and 65 years old and had the mental capacity to give informed consent. For adults with a 22q11.2 CNV, a confirmed 22q11.2 CNV diagnosis was established by fluorescence *in situ* hybridization, chromosomal microarray or multiplex ligation-dependent probe amplification. Exclusion criteria for all groups were a history of severe psychiatric or neurological disorders, substance abuse and participation in another study involving radiation exposure within the past year. All study participants were free of medication known to influence radiotracer binding at the time of scanning.^26^ One participant with 22q11 deletion was receiving aripiprazole at the time of DAT imaging and had a history of quetiapine and citalopram use. In addition, one participant in the duplication group had a history of methylphenidate (immediate- and extended-release formulations) use, and sertraline use was discontinued 2 days prior to DAT imaging (current and past psychotropic medication use is reported in Supplementary Table 1). Additionally, all participants were instructed to refrain from alcohol and nicotine in the 24 h prior to the DaT-SPECT scan. All participants gave written informed consent.

### DaT-SPECT scan

The SPECT scan was performed on a brain-dedicated SPECT system at the Academic Hospital of the University of Amsterdam (InSPira HD, Neurologica, Boston, USA). Three hours prior to the SPECT scan, participants received an injection of ∼111 MBq [^123^I]N-ω-fluoropropyl-2β-carbomethoxy-3β-nortropane ([^123^I] FP-CIT) (GE HealthCare). To protect the thyroid gland and following the standard procedure, 1 h prior to injection, participants orally received 100 mg potassium iodine to block the uptake of free iodide by the thyroid. An [^123^I]FP-CIT SPECT scan was performed to investigate DAT binding ratios (non-displaceable binding potential; BP_ND_) in the striatum.^27^ Image acquisition and processing were done as described previously.^28^ Specific striatal binding ratios were calculated automatically using the Brain Registration & Analyses Software Suite (BRASS) from HERMES Medical Solutions (Sweden).^29^ The binding ratios were calculated for the right and left striatum separately and for the whole striatum, using the occipital lobe as reference region. For the main analyses, we used the average striatal BP_ND_, defined as the mean of the left and right striatum, since we found no statistically significant differences between both sides in any of the three groups under study (Supplementary Table 2).

### MRI scan

A subset of study participants underwent magnetic resonance imaging (MRI), including four individuals with a 22q11.2 deletion, seven with a 22q11.2 duplication and all eight healthy controls. Structural MRI was not obtained for all participants due to contraindications, such as (metal) implants from congenital heart disease surgery, which are relatively common in individuals with a 22q11.2 deletion. All MRI measurements were performed on a MAGNETOM 7T MRI scanner (Siemens Healthineers, Erlangen, Germany). Striatal volume was calculated for the available scans as described in the Supplementary Methods.

### Statistical analyses

Statistical analyses were conducted in R, version 4.3.1 (R Core Team, 2023). Due to small sample sizes and non-normal distributions, non-parametric methods were used throughout. Group differences in demographics and clinical characteristics were assessed with chi-square tests or Wilcoxon rank-sum tests, as appropriate.

The primary analyses were performed using a Freedman–Lane permutation analysis of covariance (ANCOVA), implemented in the permuco R package,^30^ with striatal DAT BP_ND_ as the dependent variable and group as the main factor of interest, with age and sex included as covariates.^31^ Statistical significance was assessed using permutation testing with 10 000 permutations, and effect sizes are reported as partial eta-squared (*η*^2^_p_). This approach is robust to small sample sizes and unequal variances while accounting for covariates.^30^ Where a significant group effect was observed, *post hoc* pairwise comparisons between groups were conducted using the same method. Analyses were repeated for striatal subdivisions (left and right caudate nucleus and putamen).

To test for potential volumetric influences, a sensitivity analysis was conducted in a subset of participants with available MRI data, including striatal volume as an additional covariate. Within-group striatal asymmetry was evaluated by comparing left and right striatal DAT BP_ND_ values using Wilcoxon rank-sum tests. All statistical tests were two-sided, with a significance threshold of *P* < 0.05. Given the exploratory nature of the study, no correction for multiple comparisons was performed.

## Results

Ten adults with a 22q11.2 deletion, nine adults with a 22q11.2 duplication and eight healthy controls were recruited for this study. One participant with 22q11.2 deletion was excluded from the analyses due to substantial head movement during the SPECT scan. No significant group differences were found for age and sex (Table 1). There were no smokers in any of the groups. As expected, intelligence quotient was significantly higher in controls compared to individuals with a 22q11.2 deletion, with 22q11.2 duplication falling in between (Table 1). Most individuals with a 22q11.2 deletion had the typical A-D low-copy repeat (LCR) deletion, and one individual carried the smaller A-B deletion. Within the 22q11.2 duplication group, four subjects had a proximal (A-D) and five a distal 22q11.2 duplication (3 E-H and 2 F-H; see Table 1 and Supplementary Fig. 1).^32^

**Table 1:** Demographics of healthy controls, 22q11.2 deletion and 22q11.2 duplication

	*HC N = 8*	*22q11.2 deletion N = 9*	*22q11.2 duplication ^a^ N = 9*	*P-value*
*Age in years, Median (IQ1–IQ3)*	33.6 (23.4–51.4)	30.5 (22.7–38.1)	51.7 (33.7–59.8)	0.130 ^b^
*Sex, F/M*	5/3	5/4	6/3	0.887 ^c^
*Ethnicity, n*	2 Black 6 White	1 Hispanic 8 White	1 Hispanic 8 White	-
*FSIQ ^d^, Median (IQ1–IQ3)*	111.0 (106.5–122.5)	78.0 (70.0–84.0)	97.0 (88.0–103.0)	<0.001
*CNV, n Proximal/distal ^e^*	N/A	9/0	4/5	-

Abbreviations: HC, healthy control; F, female; FSIQ, full-scale intelligence quotient; M, male; CNV, copy number variant; N/A, not applicable.

^a^Two-family pairs were included; two sisters and a father and daughter.

^b^Kruskal–Wallis test.

^c^Pearson’s chi-squared test.

^d^Two participants with 22q11.2 deletion and one with 22q11.2 duplication completed the WAIS–IV; all others completed the short Dutch version of the WAIS–III.

^e^Proximal = including low-copy repeat (LCR) A-B; distal = excluding LCR A-B. Details about the distal 22q11.2 duplications can be found in Supplementary Fig. 1.

### DaT-SPECT

Striatal BP_ND_ are shown in Fig. 1 (individual data in combination with CNV breakpoint classification in Supplementary Fig. 1). Median BP_ND_ values, with interquartile ranges in parentheses, were 4.05 (3.78–4.64) for 22q11.2 deletion, 3.31 (3.26–4.67) for controls and 3.68 (3.11–3.91) for 22q11.2 duplication. Freedman–Lane permutation ANCOVA, controlling for age and sex, showed that median striatal DAT BP_ND_ was significantly higher in 22q11.2 deletion compared with 22q11.2 duplication with a large effect size (*F*(1,14) = 5.98, *P* = 0.026, *η*^2^_p_ = 0.30), but did not differ significantly between controls and 22q11.2 duplication (*F*(1,13) = 1.26, *P* = 0.277, *η*^2^_p_ = 0.088), nor between 22q11.2 deletion and controls (*F*(1,13) = 0.35, *P* = 0.568, *η*^2^_p_ = 0.026). Excluding the data of the participant that used aripiprazole did not change this main finding (deletion versus duplication, *F*(1,13) = 4.67, *P* = 0.049, *η*^2^_p_ = 0.26).

**Figure 1:**
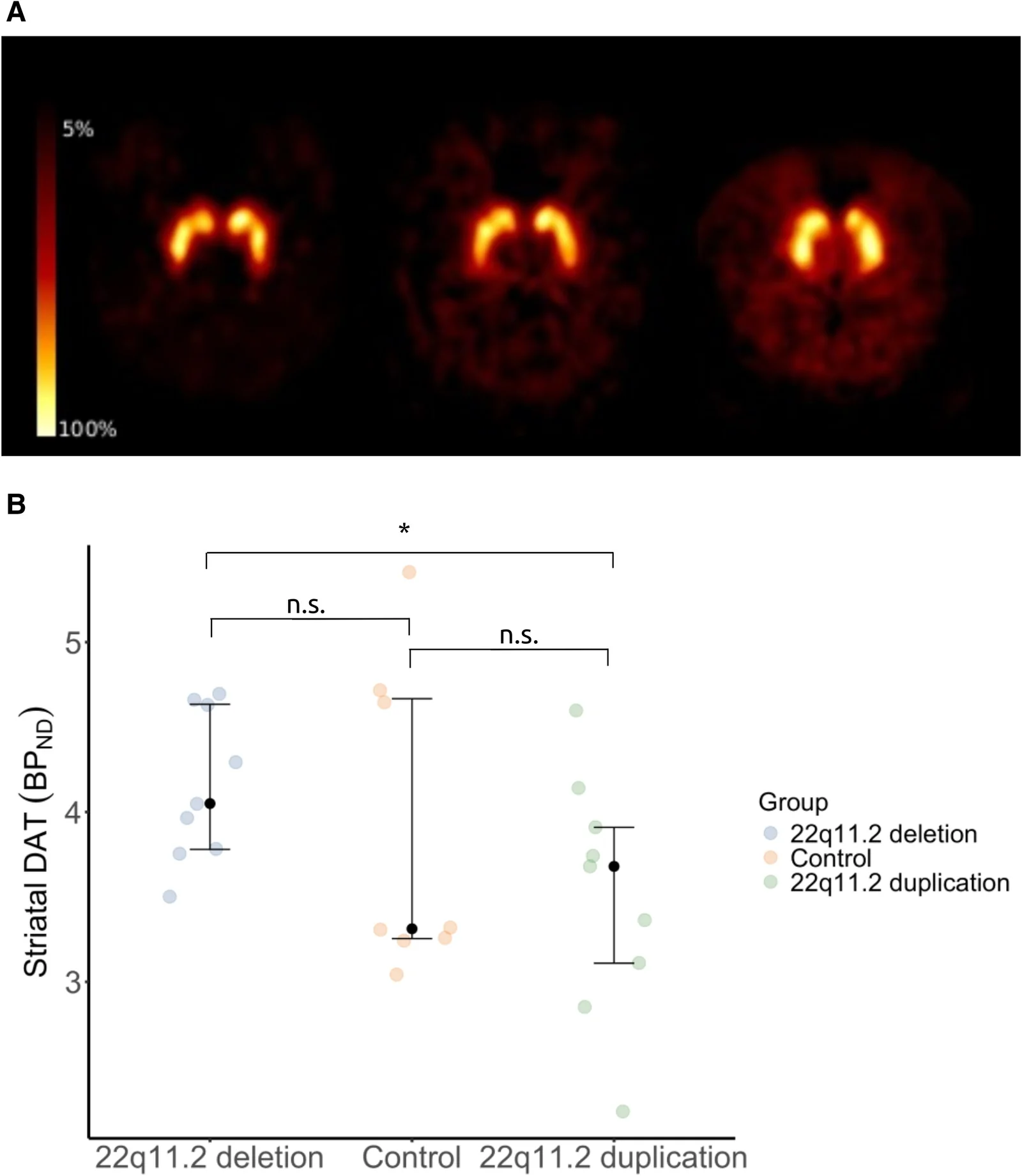
**Striatal DAT availability in 22q11.2 deletion, 22q11.2 duplication and healthy controls. (A)** Striatal dopamine transporter (DAT) availability, measured with [^123^I]FP-CIT SPECT, in a 22q11.2 deletion subject (male, 38 years), healthy control (male, 39 years) and 22q11.2 duplication subject (male, 40 years). Presented are transversal slices through the striatum. Please note that in all three subjects there is symmetric and clear uptake in the caudate nucleus and putamen. The striatal DAT availability is lowest in the 22q11.2 duplication subject, which is reflected by clearer visualization of activity outside the striatum (lowest striatal-to-non-specific binding ratio). (**B)** Striatal DAT BP_ND_ in the three study groups. Individual participant data are displayed. Central black dots indicate the median, and error bars represent the interquartile range. Group differences were tested using a Freedman–Lane permutation ANCOVA adjusting for age and sex. A significant difference was observed between 22q11.2 deletion and 22q11.2 duplication (*F*(1,14) = 5.98, *P* = 0.026, *η*^2^p = 0.30), while all other comparisons were not significant (n.s.). Significant differences are denoted by an asterisk (*P* < 0.05). Abbreviations: DAT, dopamine transporter; BP_ND_, non-displaceable binding potential; n.s., not significant.

Sex emerged as a significant covariate in models involving 22q11.2 duplication (*P* ≤ 0.008) and was marginally significant in the comparison between controls and 22q11.2 deletion (*P* = 0.054). Age was a significant covariate only in the comparison between controls and 22q11.2 deletion (*P* = 0.035). Visual inspection of age-related patterns (Fig. 2) suggested that the 22q11.2 deletion and 22q11.2 duplication groups did not exhibit the same age-related decline observed in controls. To formally evaluate this, we tested the age × group interaction: this effect was not significant (*P* = 0.426).

**Figure 2:**
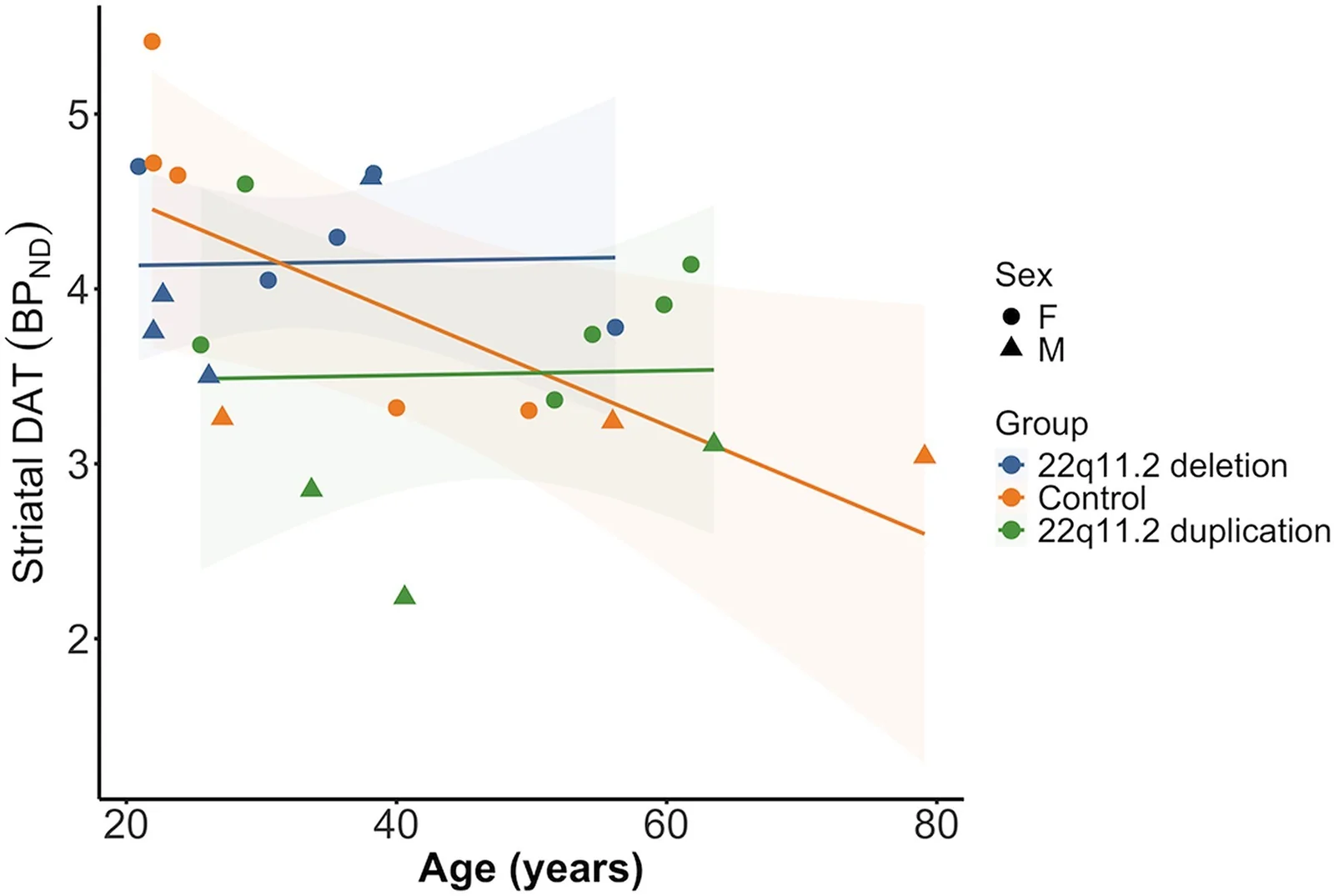
**Association between age and striatal DAT availability.** Scatterplots show individual striatal DAT BP_ND_ as a function of age, stratified by group. Each point represents a participant; males are indicated by triangles and females by circles. Solid lines represent group-specific linear regression fits with shaded areas denoting 95% confidence intervals. Regression lines were fitted per group and are independent of sex, which is shown for visualization only. The age × group interaction was tested using a Freedman–Lane permutation ANCOVA adjusting for sex and was not significant (*F*(2,18) = 0.890, *P* = 0.428). Abbreviations: BP_ND_, non-displaceable binding potential; DAT, dopamine transporter; F, female; M, male.

No significant asymmetry between median left and right striatal DAT BP_ND_ was observed within any group (Supplementary Table 2). DAT BP_ND_ was also compared between subdivisions of the striatum, comprising the left and right caudate nucleus and putamen. In all structures, but left caudate nucleus, the difference between 22q11.2 deletion and 22q11.2 duplication remained significant (Supplementary Table 3).

To assess the potential influence of striatal volume, the main analyses were repeated with striatal volume included as an additional covariate. Striatal DAT BP_ND_ remained significantly higher in 22q11.2 deletion compared with 22q11.2 duplication with a large effect size (*F*(1,14) = 6.78, *P* = 0.038, *η*^2^_p_ = 0.30), but did not differ significantly between controls and 22q11.2 duplication (*F*(1,13) = 1.26, *P* = 0.277, *η*^2^_p_ = 0.088), nor between 22q11.2 deletion and controls (*F*(1,13) = 0.35, *P* = 0.568, *η*^2^_p_ = 0.026).

## Discussion

In this study, we compared striatal DAT availability across adults with 22q11.2 deletions and duplications, and healthy controls. The deletion group showed significantly higher median DAT availability than the duplication group. However, no significant differences were found between the 22q11.2 CNV groups and healthy controls. Because of the small sample size, these results should be interpreted with caution, and larger studies are needed to reproduce our findings and to clarify whether more subtle differences are present.

These findings may reflect an effect of 22q11.2 CNVs on presynaptic striatal dopaminergic regulation and a gene-dosage effect. Such a gene-dosage effect, as previously been demonstrated for *COMT* at the mRNA level in individuals with a 22q11.2 deletion and a 22q11.2 duplication^19^ may contribute to these dopaminergic differences, although this interpretation remains speculative. Increased DAT availability in individuals with a 22q11.2 deletion aligns with prior neuroimaging studies examining presynaptic dopaminergic function in this population. Increased striatal vesicular monoamine transporter binding, like DAT also a presynaptic marker, has been reported in adults with a 22q11.2 deletion (mean age 41.5 ± 8.7 years) compared with healthy controls using positron emission tomography (PET).^11^ Consistent with these reports, increased striatal dopamine synthesis capacity has also been observed in 22q11.2 deletion (mean age 26.1 ± 7.7 years) compared with both healthy controls and 22q11.2 duplication using [^18^F] dihydroxyphenylalanine (DOPA) PET imaging.^18^

Striatal dopamine signalling is further shaped by local GABAergic and cholinergic networks, as well as glutamatergic afferents from cortical and thalamic pathways.^33^ Beyond *COMT*, several other genes within the 22q11.2 region may contribute to dopaminergic modulation. One example is *proline dehydrogenase* (*PRODH*), which is involved in glutamate metabolism and may thereby indirectly influence dopamine signalling, and possibly DAT expression.^34,35^ Additionally, *DiGeorge syndrome critical region gene 14* (*DGCR14* in humans; *Dgcr14* in mice) has been associated with reduced protein kinase R-like endoplasmic reticulum kinase expression, leading to impaired endoplasmatic reticulum stress tolerance and abnormal filamentous actin (F-actin) dynamics, as demonstrated in dopaminergic neurons derived from induced pluripotent stem cells (iPSCs) of patients with 22q11.2 deletion and in a mouse model.^36^

Genes outside the 22q11.2 locus may further influence dopaminergic balance. One example is *dystrobrevin-binding protein 1* (*DTNBP1*; dysbindin), located on chromosome 6, which has been linked to dopaminergic function and cognition. Previous studies have reported that concomitant reductions in *COMT* and *DTNBP1* expression in individuals with a 22q11.2 deletion are associated with cognitive disturbances that are not explained by alterations in either gene alone.^37^ In mice, reductions in *Comt* and *Dtnbp1* produce a hypoactive mesocortical and hyperactive mesostriatal dopamine pattern, consistent with the elevated striatal DAT availability observed in the current study, and may help partly explain the co-occurrence of cognitive difficulties and psychosis risk in 22q11.2 deletion.^37^

Taken together, these findings highlight the complexity of striatal dopamine regulation in 22q11.2 CNVs, likely reflecting interactions between presynaptic dopaminergic mechanisms, broader neurotransmitter systems and genetic modifiers within and beyond the 22q11.2 gene region. Future studies combining DaT-SPECT with complementary PET tracers targeting different aspects of dopamine function (e.g. D_2/3_ receptors, endogenous dopamine release) may help further disentangle these mechanisms.

### Interindividual variability in DAT availability: effects of age and sex

The absence of a significant difference between 22q11.2 deletion and healthy controls may reflect greater heterogeneity within the control group, including a wider age range. This heterogeneity together with a small sample size could have reduced statistical power to detect more subtle group differences with controls. Consistent with this, substantial interindividual variability in striatal DAT availability among healthy individuals has been consistently reported in the literature, with effects of both age and sex on DAT availability.^31,38^ In the current study, sex emerged as a significant covariate in models involving 22q11.2 duplication and was marginally significant in comparisons between controls and 22q11.2 deletion, with female sex showing a positive effect on DAT availability. Supporting these findings, higher urinary dopamine levels have been reported in females with 22q11.2 deletion compared to males.^39^ This may reflect the sexually dimorphic activity of COMT, in part due to oestrogen-mediated downregulation of the enzyme.^40^

### Age-related DAT trajectories

To further characterize interindividual variability, striatal DAT availability was examined as a function of age. In healthy controls, DAT availability showed a clear negative association with age, consistent with previously reported age-related decreases of 4–6.7% per decade.^38^ In individuals with 22q11.2 CNVs, no significant association between age and striatal DAT availability was observed; however, given the limited sample size, the absence of an association should be interpreted with caution.

In a related context, attenuated iron accumulation in the basal ganglia was reported in young adults with a 22q11.2 deletion (mean age 23.61 ± 3.87 years) compared to controls.^41^ Attenuated iron accumulation in 22q11.2 deletion may therefore reflect altered basal ganglia maturation or dopaminergic development, further supporting the notion of a divergent maturational trajectory in this population. Whether this divergent trajectory persists into later adulthood remains unclear, as data beyond 30 years are limited. Larger longitudinal studies will be required to determine whether these trajectories remain stable over time. This is relevant given that multicentre data showed an increased risk of developing Parkinson’s disease in individuals with a 22q11.2 deletion aged ≥40 years, with a cumulative risk of 14% in those aged ≥50 years.^9^

Since Parkinson’s disease is characterized by nigrostriatal dopaminergic degeneration and reduced striatal DAT binding, one might predict lower striatal DAT binding in at-risk groups.^42^ In this context, it is of interest that Harraz and co-workers proposed that initial high DAT expression per neuron may increase vulnerability to degeneration in Parkinson’s disease later in life.^43^ DAT facilitates synaptic dopamine reuptake, and excessive reuptake can lead to increased dopamine oxidation and neurotoxicity of dopaminergic neurons, contributing to neuronal loss.^43^ According to the auto-toxicity theory, toxic products of spontaneous and enzymatic oxidation of cytoplasmic catecholamines (e.g. dopamine) affect neuronal integrity,^44^ potentially causing the death of catecholamine-containing neurons.^44^ Taken together, an initial hyperdopaminergic state (characterized by high DAT expression) might eventually cause auto-neurotoxicity and subsequent loss of dopaminergic neurons (and DAT), a mechanism that may contribute to the increased risk of Parkinson’s disease in 22q11.2 deletion.

### Clinical implications for DaT-SPECT imaging

Understanding striatal DAT availability in neurologically healthy individuals with a 22q11.2 deletion is crucial for interpreting DaT-SPECT imaging when evaluating 22q11.2 deletion patients with parkinsonism. In this study, we found that in 22q11.2 deletion without a history of a neurological disorder, median striatal DAT binding values were not decreased compared to controls, and the putamen and caudate ratios did not differ compared to controls. Notably, a detailed characterization of Parkinson’s disease in 22q11.2 deletion reported an association of history of antipsychotic treatment with a significantly delayed Parkinson’s disease diagnosis compared with antipsychotic-naive patients (median 5 versus 1 year), highlighting how psychiatric comorbidity and particularly its treatment with antipsychotics may obscure early Parkinson’s disease recognition in this population.^25^ Notably, 22q11.2 deletion confers one of the highest known genetic risks for schizophrenia, with ∼25% of individuals developing a psychotic disorder in adulthood.^2,45^ Interestingly, DAT imaging can be helpful to differentiate between Parkinson’s disease and neuroleptic-induced parkinsonism also in 22q11.2 deletion patients with parkinsonism suggestive of Parkinson’s disease.^25^ Finally, the association between 22q11.2 deletion and Parkinson’s disease may be relevant also for schizophrenia. Epidemiological studies indicate that individuals with schizophrenia have an ∼2-fold increased risk of developing Parkinson’s disease in the general population.^46^ Furthermore, genetic studies have identified shared polygenic risk loci between schizophrenia and Parkinson’s disease^47^ suggesting a common biological vulnerability that is at least partially independent of antipsychotic treatment.

### Limitations

Several limitations should be acknowledged. First, given the cross-sectional study design, we could not determine when or if alterations in striatal DAT occur or whether these changes remain stable over time in 22q11.2 CNV cases. Longitudinal studies are therefore needed to replicate our findings and to assess whether striatal DAT availability may predict progression to Parkinson’s disease. The relatively young age of the cohort, particularly among individuals with a 22q11.2 deletion, may allow for future follow-up, as clinical phenotypes may still evolve in this high-risk population.

Moreover, one participant with 22q11.2 deletion was using aripiprazole at the time of DAT imaging. However, excluding this participant from the analysis did not change the main findings. Additionally, one participant with 22q11.2 duplication was treated with sertraline (75 mg), which was discontinued 2 days prior to the SPECT scan. The same participant had a history of methylphenidate use (immediate- and extended-release formulations) and a diagnosis of ADHD. Previous studies have suggested that ADHD and methylphenidate use may influence *in vivo* DAT binding. However, in this case exposure was limited to 8 months, with the last intake occurring 10 years prior to assesment^48^ (Supplementary Table 1). Nevertheless, comorbidity with neuropsychiatric disorders like anxiety or ADHD and long-term treatments with psychopharmaceuticals are common in individuals with 22q11.2 CNVs, which complicates studies in these individuals. Therefore, larger studies, ideally with a longitudinal design, are needed to better understand differences in DAT binding between 22q11.2 deletion and duplication.

Finally, this study focused on a relatively healthy subgroup of individuals with 22q11.2 CNVs who had no current psychiatric and Parkinson’s disease diagnosis and were not using psychotropic medication. Although this limits generalization to the broader 22q11.2 CNV population, it reduced potential confounding effects of psychiatric symptoms and medication on DAT tracer binding,^26^ allowing for a clearer interpretation of dopaminergic alterations attributable to genetic variation. Lastly, this sample included five individuals with a distal 22q11.2 duplication (distal to the 22q11.21 microdeletion syndrome region), which may have led to an underestimation of a potential gene-dosage effect.

## Conclusion

In this study, adults with a 22q11.2 deletion exhibited higher median striatal DAT availability compared with adults with a 22q11.2 duplication, consistent with a potential gene-dosage effect on dopaminergic regulation. Both deletion and duplication groups showed no age-related decline in DAT availability, suggesting that these CNVs may influence the trajectory of striatal dopamine function over time. Larger longitudinal studies will be essential to confirm these observations, clarify age-related dopaminergic changes, and determine to what extent striatal DAT availability changes over time may predict later neuropsychiatric or neurodegenerative outcomes in individuals with 22q11.2 CNVs.

## Supplementary Material

fcag395_Supplementary_Data

## Data Availability

The data that support the findings of this study are not publicly available due to privacy and ethical restrictions and are available from the corresponding author upon reasonable request. The code used for data analysis is publicly available at: https://github.com/JeltjeSpapens/SPECT-22q11-CNV.git.
