# Supplementary material for: Striatal dopamine transporter binding in adults with 22q11.2 copy number variants: a SPECT study
Source: Brain Commun. 2026 Oct 19;8(6):fcag395. doi: 10.1093/braincomms/fcag395 (PMC13725163; doi:10.1093/braincomms/fcag395)
Supplement: Supplementary file 1 [file fcag395_supplementary_data.zip › Supplementary_material.docx]

**Supplementary Material**

**Supplementary methods**

*Calculation of striatal volume from MRI*

A whole-brain MP2RAGE (Magnetization Prepared 2 Rapid Acquisition Gradient Echoes) scan was performed at the beginning of the scanning session ^1^. T1w anatomical images were pre-processed using a Presurfer MATLAB script (MathWorks, Natrick, MA) which performed de-noising, brain extraction, and intensity normalization ^2^. Subsequently, FreeSurfer’s recon-all pipeline was performed to obtain striatal volume measures using the ASEG segmentation output, analysed separately for the left and right caudate nucleus and putamen.

**Supplementary Table 1. Medication**

|  | **Current/past** | **Medication** | **Dose** | **Duration** | **Indication** |
| --- | --- | --- | --- | --- | --- |
| Participant 1  22q11 duplication ^1^ | Current^3^ | Sertraline | 75 mg | 6 months | Anxiety symptoms |
|  | Past | Ritalin | 15 mg | 5 months | ADHD |
|  | Past | Concerta | 18 mg | 3 months | ADHD |
|  | Past | Amitriptyline | 10 mg | 16 months | Nerve pain |
|  | Past | Topiramate | 100 mg | 9 months | Migraine |
|  | Past | Sumatriptan | 50-100 mg | 36 months | Headache |
|  | Past | Frovatriptan | 2,5 mg | 3 months | Migraine |
|  | Past | Pregabalin | 150 mg | 10 months | Nerve pain |
| Participant 2  22q11 deletion^2^ | Current | Aripiprazole | 5-10 mg | 12 months | History of psychotic symptoms |
|  | Past | Seroquel | 50 mg | 96 months | Psychotic symptoms |
|  | Past | Citalopram | 8 mg | ? | Mood |

^1^Previous diagnosis of ADHD. Ritalin and Concerta were used more than 10 years ago.

^2^Previous diagnoses of depression, anxiety, and autism.

^3^Stopped 2 days before the scan

**Supplementary Table 2. Striatal DAT SPECT asymmetry**

| **BP_ND_** | **Left striatum** | **Right striatum** | **p-value** ^a^ |
| --- | --- | --- | --- |
| Healthy control, N=8 | 3.46 (3.32- 4.74) | 3.26 (3.14- 4.59) | 0.080 |
| 22q11.2 deletion, N=9 | 3.94 (3.69– 4.62) | 4.16 (3.88– 4.54) | 0.155 |
| 22q11.2 duplication, N=9 | 3.60 (3.09– 3.84) | 3.64 (3.13– 4.00) | 0.636 |

Abbreviations: BP_ND_, non-displaceable binding potential; DAT, dopamine transporter; SPECT single photon emission computed tomography.

IQR, inter quartile range. Data is presented as median (IQR).

^a^ Wilcoxon signed-rank test comparing left vs. right striatum within each group.

**Supplementary Table 3. Striatal DAT SPECT analyses in subdivisions**

| **BP_ND_** | **Healthy control**  N=8 | **22q11.2 deletion** N=9 | **22q11.2**  **duplication** N=9 | **Comparisons** | **p-value** ^a^ | **η^2^_p_** |
| --- | --- | --- | --- | --- | --- | --- |
| Left caudate nucleus | 3.60  (3.49-5.06) | 4.20  (3.94-4.81) | 3.81  (3.18-4.26) | DEL – HC  DUP – HC  DEL – DUP | 0.957  0.200  0.073 | <0.001  0.125  0.214 |
| Right caudate nucleus | 3.46  (3.93-4.82) | 4.19  (3.96-4.47) | 3.68  (3.30-4.19) | DEL – HC  DUP – HC  DEL – DUP | 0.671  0.349  **0.015** | 0.014  0.067  0.345 |
| Left putamen | 3.52  (3.18-4.36) | 3.77  (3.53-4.09) | 3.36  (2.99-3.37) | DEL – HC  DUP – HC  DEL – DUP | 0.781  0.127  **0.034** | 0.006  0.168  0.232 |
| Right putamen | 3.13  (2.98-4.32) | 4.12  (3.85-4.62) | 3.49  (2.94-3.84) | DEL – HC  DUP – HC  DEL – DUP | 0.175  0.779  **0.035** | 0.138  0.006  0.276 |

Abbreviations: DAT, dopamine transporter; SPECT single photon emission computed tomography; BP_ND_, non-displaceable binding potential; HC, healthy control; DEL, Deletion, 22q11.2 deletion; DUP, 22q11.2; IQR, inter quartile range.

Data is presented as median (IQR) and significant p-values in bold.

^a^ Group differences were assessed with a Freedman Lane permutation ANCOVA correcting for age and sex.

**Supplementary Figure 1. Striatal DAT binding for the whole striatum**

Striatal DAT BP_ND_ in individual participant data displayed. Distal 22q11.2 duplications: 3 individuals with E-H low copy repeat duplications and 2 individuals with F-H duplications. Abbreviations: DAT, dopamine transporter; BP_ND_, non-displaceable binding potential.

**Supplementary material reference list**

1.	Marques JP, Kober T, Krueger G, van der Zwaag W, Van de Moortele P-F, Gruetter R. MP2RAGE, a self bias-field corrected sequence for improved segmentation and T1-mapping at high field. NeuroImage. 2010/01/15/ 2010;49(2):1271-1281. doi:<https://doi.org/10.1016/j.neuroimage.2009.10.002>

2.	Kashyap S. srikash/presurfer: ondu (1.0). Zenodo. <https://doi.org/10.5281/zenodo.4626841>
